# Supplementary material for: Are carbon emissions trading and green financial instruments synergistic? -Comprehensive quantitative research based on content analysis
Source: PLoS One. 2024 Mar 7;19(3):e0298601. doi: 10.1371/journal.pone.0298601 (PMC10919601; doi:10.1371/journal.pone.0298601)
Supplement: S1 Data — (ZIP) [file pone.0298601.s001.zip › Data/地级市Z_F工作报告.zip!/╕ú╜¿/─■╡┬╩╨/└┤╫╘╣1⁄2╓┌║┼í╛┬φ┐╦╩2╛▌═°í┐_2009.docx]

各位代表：

现在，我代表市人民政府向大会报告政府工作，请予审议，并请市政协各位委员和其他列席人员提出意见。

一、2008年的主要工作

2008年，在省委、省政府和市委的坚强领导下，全市各级政府认真贯彻党的十七大、十七届三中全会精神，全面落实科学发展观，按照省委“四求先行”和对宁德提出“拓展一线、壮大闽东北一翼”的要求，积极应对宏观经济环境急剧变化的严峻挑战，奋力攻坚克难，扎实有效作为，全力推进环三都澳区域发展和海西东北翼中心城市建设，全市经济社会持续较快发展，实现了市二届人大三次会议确定的各项目标。其中，财政总收入、城镇登记失业率、森林覆盖率、新型农村合作医疗参保率和城镇职工养老保险参保人数5项指标提前实现“十一五”规划预期目标。

（一）国民经济持续较快增长。全市生产总值543亿元，比上年增长14.6%，增幅居全省第2位。三次产业增加值分别增长6.5%、20.3%、12.7%，比重调整为19.8:40.4:39.8。财政总收入突破40亿元，增长19.7%，地方级财政收入24亿元，增长20%，均比“十五”末翻一番。城镇居民人均可支配收入13936元，实际增长7.4%；农民人均纯收入5404元，实际增长10.2%，增幅居全省首位，创1998年以来新高。各项人民币存、贷款余额分别为365亿元和398亿元，分别增长24.9%和25.6%。城镇登记失业率3.77%，人口自然增长率6.3‰。

（二）环三都澳区域发展效应扩大。省委、省政府先后专题研究了《环三都澳区域发展规划》，并于去年9月由省政府批复实施，这是我市在省委、省政府层面研究的第一个综合性区域发展规划。与总体规划相衔接的分区规划和各专项规划修编工作扎实开展。启动区溪南半岛开发取得突破性进展，省政府与中海油正式确定在溪南半岛共同开发建设海西宁德工业区，正抓紧组建工业区管理机构和合作开发公司，盐田至溪南疏港公路加快建设，沈海高速公路湾坞至溪南连接线、溪南220千伏变电站和双回路输变电线路、杯溪流域至溪南半岛供水工程等基础设施项目前期工作有序推进。核电、火电、电机、修造船等一批重大项目加快向环三都澳区域集聚，去年“6·16”宁德投洽会上，环三都澳区域签约项目61个，投资金额占全市签约项目投资总额的76.8%，其中投资额超10亿元的大项目8个，总投资达260亿元。

（三）社会主义新农村建设扎实推进。全市农林牧渔业总产值187亿元，增长7%，增幅居全省首位，创1998年以来新高。新农村试点示范村建设有序推进，落实各类帮扶资金4715万元，实施帮扶项目1008项。各项强农惠农政策有效落实，粮食生产稳中有升，全市粮播面积199万亩、粮食产量64万吨；茶叶、食用菌、水产等主导产业不断发展壮大，福鼎、蕉城分别被评为“中国名茶之乡”、“中国大黄鱼之乡”，福安成为我省首个全国绿色食品（茶叶）原料基地市，福鼎被农业部评为“白茶加工基地和产业基地”，“古田银耳”获得中国驰名商标，“坦洋工夫”获得地理标志保护产品及证明商标注册证书，10家企业入选2008年度中国茶叶行业百强企业，21家农业企业被确认为省级以上农业产业化重点龙头企业。农业标准化步伐加快，建成8个国家级农业标准化示范区，新增绿标产品5个、无公害产品7个。农村基础设施逐步完善，投资17.2亿元，完成建制村通村公路硬化2435公里，累计通建制村2132个，分别占任务数的99.4%、99%，新建6个农村客运站和234个农村候车亭，提前两年超额完成省委、省政府下达的“年万里”农村路网工程任务，兑现了对全市人民的庄重承诺；提前全面完成广播电视村村通任务，完成农村水利“六千工程”投资6540万元，完成31个乡镇343个村垃圾治理任务，新建6000口农村户用沼气池。

（四）工业经济持续快速增长。全市工业总产值643亿元，增长26.1%。其中，规模以上工业完成产值522亿元，增长31.5%，规模以上工业增加值140亿元，增长25.8%，增幅均连续四年居全省首位。临海产业加快发展，福建省第一个核电站——宁德核电去年2月正式动工，一期工程一、二号机组进入主体土建施工阶段，国家能源局同意核电送出工程开展前期工作；大唐火电二期2号机组已投产发电，三期工程前期工作顺利有序推进；聚合物锂离子电池、福建东元电机、大型钢铁等项目前期工作继续推进。电机电器、船舶修造、食品加工、汽摩配件等传统优势产业逐步壮大，完成产值321亿元，增长21.8%，其中电机电器产业入选中国百佳产业集群。新增规模以上企业171家，新增投资千万元以上工业新增长点项目84个，全年完成工业投资107亿元，增长39.5%；福安被授予“中国中小电机出口基地”称号，福鼎申报“中国化油器名城”通过中国机械工业联合会评审，“安波”电机被认定为中国驰名商标，28家企业28种产品获得省级名牌产品。节能减排力度加大，全年单位生产总值能耗降低0.7%，化学需氧量减排1.5%。

（五）投资拉动强劲有力。全社会固定资产投资237亿元，增长30.7%，拉动经济增长7.7个百分点。68个重点在建项目完成投资121亿元，增长78.6%。温福铁路宁德段铺轨全面展开，我市第一条东西走向的高速公路——宁武高速公路宁德段开工建设，宁古二级公路加快建设；宁德洪口水电站1号机组、寿宁刘柴和下东溪水电站实现投产发电，220千伏霞浦岚后、福鼎桐城二期、宁德兰田二期输变电工程建成投产；衢宁铁路、京福铁路古田段、白马港铁路支线、东南沿海货运铁路、沈海高速公路复线、京台高速公路、福泰高速公路、滨海大道等前期工作加快推进。宁德会展中心、宁德市污水处理厂、东侨科技大楼、三都澳迎宾馆、东侨中学教学楼建成投入使用；宁德学院学科院系楼、学生食堂和宿舍楼已开工建设；宁德职业技术学院、宁德市医院、宁德市中医院病房大楼、宁德垃圾焚烧厂等一批项目加快推进。

（六）消费需求大幅上升。全社会消费品零售总额199亿元，增长22.1%，增幅创1996年以来新高。居民消费价格指数104.1。旅游业发展持续加快，全年接待游客550万人次，旅游收入41亿元，分别增长17.4%和19.6%，宁德世界地质公园被列入向联合国教科文组织推荐名单，白水洋、鸳鸯溪、太姥山被评为国家4A级景区。商贸流通业加快发展，中国海峡大茶都一期工程落成开业；古田食用菌批发市场物流配送中心建成投入使用，柘荣太子参专业批发市场、福鼎闽浙边界粮油批发市场、蕉城华洲水产品批发市场加快建设；改造农贸市场11个，建成生猪、蔬菜等副食品基地38个、“农家店”150个。产品质量和食品药品安全专项整治深入开展，及时开展乳制品质量安全专项清查，积极应对问题奶粉事件影响，制假售假、商业欺诈等违法犯罪活动有效遏制。

（七）改革开放富有成效。行政审批制度改革持续深化，市行政服务中心迁入新址，49个单位进驻中心，实现行政审批事项“只进一家门、服务一条龙”。乡镇机构、集体林权、海域使用权、小型水利设施产权等各项改革取得明显成效。国有资产监督管理体系逐步完善，财政、金融、流通、电力、城市管理、社会事业等体制改革扎实推进。对外开放持续拓展，成功举办第九届宁德投洽会，积极参加第十二届中国投洽会等重大招商活动，全年新批外商投资项目19项，合同外资1.38亿美元，实际利用外商直接投资（验资口径）3814万美元，增长76%，增幅居全省首位。机电、船舶、水产等重点行业出口持续增长，全年外贸出口总额8.2亿美元，增长43.1%，增幅居全省第2位。对台合作交流持续扩大，台湾民俗文化进香团以直航方式抵达宁德，实现了宁台客运直航的历史性突破；宁德港被列入两岸海运直航港口，基隆市市长访问我市，并与我市签订了《建立紧密经济文化合作关系的协议》，两地将从港口经济、海上客货直航、招商会展、渔业、旅游业、民间信仰交流、民间团体交往交流等方面建立紧密合作关系。

（八）民生保障逐步完善。全市21个23项为民办实事项目取得实效，其中20项全面完成，3项跨年度项目达到即期目标。全年新增城镇就业2.49万人、下岗失业人员再就业1.04万人、农村劳动力转移就业5.1万人。社会保障体系逐步完善，城乡低保对象达14万人，全年参加城镇职工医疗、养老、失业、工伤、生育保险的职工分别达到20.1万人、20万人、11.6万人、10.8万人、6.4万人，解决医改前关闭破产企业退休人员参加城镇职工基本医疗保险1.34万人。“造福工程”和滑坡险情村搬迁5691户25062人，完成灾后重建223户，动工建设经济适用房项目8个，新增供应解决中低收入家庭住房困难户832户，新增发放城市低保家庭住房租赁补贴578户，参加农房保险71.75万户，新建“侨居造福工程”住房120套。发放库区移民后期扶持资金1.06亿元。新建、改扩建农村敬老院11所。殡葬改革取得新突破，殡葬执法和清理坟墓工作力度进一步加大，全市平均火化率达74%，提高27.9个百分点，首次超额完成省定指标。支援四川地震灾区抗震救灾和恢复重建工作扎实开展，累计募集捐款5801万元，第一批援建的彭州市升平镇场镇道路项目建成投入使用，彭什公路升平段改造、文化站、幼儿园等项目按计划有序推进。

（九）社会事业不断进步。科技创新能力不断提高，与省科技厅建立厅市科技工作会商制度，成立市高新技术创业服务中心、市电机工程研究院和8个研发中心，霞浦、柘荣通过国家科技进步县考核；设立宁德市院士科技园，聘请18位“两院”院士和专家为市政府顾问。城乡义务教育不断巩固提高，改造农村中小学D级危房5.3万平方米，建成农村寄宿制学校27所；义务教育阶段“两免一补”政策全面落实，幼儿入园率达86.62%，高中阶段毛入学率达66.06%，中等职业教育资源整合进展顺利。文化事业加快发展，建成闽东苏区纪念馆、闽东畲族文物展馆和蔡威事迹展览馆，11个项目入选国家级非物质文化遗产名录，新建、扩建乡镇综合文化站37个、“农家书屋”170个，在全省率先完成农村电影放映任务。第十二届全民健身节成功举办，建成农民体育健身工程208个，宁德籍运动员在国际、全国比赛中获得5金1银的好成绩。大力弘扬闽东精神，公民思想道德教育、社会主义核心价值观教育、未成年人思想道德教育不断强化，各类精神文明创建活动深入开展，市民文明素质不断提高。公共卫生体系和疾病防控体系基本形成，应对突发公共卫生事件能力不断提高，新型农村合作医疗参合率达86.32%，参加城镇居民基本医疗保险36万人。低生育水平保持稳定，连续15年完成省下达的人口控制目标，古田县获得全国计生优质服务先进县称号，5个县（市、区）被评为省级计生优质服务先进县。土地、矿产资源保护力度加大，连续10年实现耕地占补平衡。人事、编制、统计、老区、民族、宗教、广播电视、新闻出版等工作取得新成效，外事、侨务、档案、老龄、地方志、库区移民、水文气象、残疾人事业、关心下一代等工作进一步加强。

（十）政府建设进一步加强。认真学习贯彻落实监督法，依法执行人大及其常委会决议，认真办理人大代表建议、意见，自觉接受人大及其常委会的法律监督和工作监督。支持人民政协履行政治协商、民主监督、参政议政职能，定期向人民政协通报工作，认真办理政协委员提案。全年办理人大代表建议199件、政协委员提案300件，办结率均为100%。加强与民主党派、工商联、无党派人士和工会、妇联、共青团等人民团体的联系，虚心听取各界人士和人民群众的意见和建议。认真贯彻依法行政实施纲要，行政执法行为和行政复议工作更加规范，“清违”工作取得阶段性成效。“五五”普法和“百千万”基层普法依法治理活动深入开展。政府运作机制更加有效，实行集中签订责任状、集中考评，市政府与各县（市、区）政府、市直有关部门签订的13个重点专项工作责任状全面完成，有力推动了政府工作落实。政府信息公开稳步推进，效能建设、绩效评估工作继续深化。廉政建设和反腐败工作不断加强，损害群众利益的突出问题得到较好治理。“平安宁德”建设实现阶段性目标，8个县（市、区）被省委、省政府命名为“平安县（市、区）”，“平安乡镇（街道）”创建率突破90%。矛盾纠纷排查调处不断深化，一批突出信访问题得到化解。安全生产监管责任有效履行，重点行业、重点领域生产安全隐患排查治理取得实效，全市安全生产形势保持稳定。应急管理体系逐步健全，应对突发公共事件能力逐步提升。国防动员、民兵预备役、双拥支前、海防、人防、反走私等工作取得新进展。

各位代表，回顾过去一年的工作，面对复杂多变的形势，我们取得的成绩来之不易。这是在改革开放30年发展的基础上取得的，是市委正确领导、科学决策的结果，是全市人民齐心协力、着力先行的结果，是社会各界人士广泛参与、大力支持的结果。在此，我代表宁德市人民政府，向辛勤工作、勇于奉献的全市人民致以崇高的敬意，向给予政府工作有力支持和有效监督的人大代表、政协委员、各民主党派、工商联、各人民团体、无党派人士、离退休老同志和社会各界人士，向为宁德发展作出积极贡献的中央及省属驻宁单位、驻宁部队、武警官兵、公安民警、民兵预备役人员，向关心支持宁德发展的港澳台同胞、海外侨胞、国际友人，表示诚挚的感谢！

在肯定成绩的同时，我们也清醒地看到，政府工作与形势的要求和人民的期望仍有差距，经济社会发展中还存在不少困难和问题：一是受世界金融危机和宏观经济环境变化的影响，部分经济指标增速趋缓，出口形势严峻，一些行业和中小企业生产经营困难，经济持续较快增长压力加大；二是财政政策性减收因素比较集中，刚性支出压力加大，将直接影响财政正常运转和收支平衡；三是资金、土地、节能减排等瓶颈制约更加突出，项目征地难、落地难问题尚未得到根本解决；四是中心城市带动能力仍然较弱，县域经济发展动力和活力不足，沿海与山区、城镇与农村差距仍然较大；五是农业基础设施仍较薄弱，农民持续增收难度较大；六是社会管理和服务水平有待提高，劳动就业、社会保障、教育事业、医疗卫生、社会治安、安全生产和食品安全等方面还存在薄弱环节；七是一些干部事业心、责任心不强，一些部门之间推诿扯皮、办事拖拉等“中梗阻”问题和一些消极腐败现象仍然存在，投资软环境还有待进一步改善。我们要在深化改革开放、持续加快发展中，解决好这些前进中的问题。

二、2009年政府工作总体目标要求和主要任务

2009年是新中国成立六十周年，也是我市深入实施“十一五”规划，全力推进环三都澳区域发展和海西东北翼中心城市建设的重要一年。我们面对难得的机遇，中央、省相继出台一系列保增长、扩内需、调结构的政策措施，国家支持海西建设、省里支持环三都澳区域发展已成为共识，为我们有效抵御风险、加快发展步伐提供了强大动力；经过改革开放30年发展，我市的综合实力和经济调节能力有了较大提高，发展的潜力正在逐步释放，特别是环三都澳区域发展战略的引领、带动效应不断扩大，宁德正处在“蓄势而发、快速发展、力求先行”的发展新平台上。同时，我们应当清醒地看到，我们也面临着严峻的挑战，国际金融危机引发的全球性经济衰退尚未见底，外部需求大量减少，各种不利因素叠加对我市的影响正逐步加大，今年是进入新世纪以来我市经济发展最为困难的一年。面对复杂多变的形势，我们既要增强机遇意识，坚定发展信心，又要增强忧患意识，保持清醒头脑，把思想和行动统一到中央对当前形势的分析判断上来，统一到中央和省委、省政府以及市委的决策部署上来，化风险为机遇，变压力为动力，坚定不移地推进全市经济社会又好又快发展。

2009年政府工作的总体要求是：以邓小平理论和“三个代表”重要思想为指导，深入学习实践科学发展观，认真贯彻党的十七大、十七届三中全会精神，按照省委八届五次全会、省十一届人大二次会议和市委二届五次全会的部署，坚持“四求先行”、“四个重在”、“四个带动”、“四重三农”，把促进经济持续较快发展作为首要任务，大力弘扬闽东精神，全力实施环三都澳区域发展战略，进一步解放思想、深化改革、扩大开放，着力扩大内需、调整结构、保障民生，着力发展农村、突出工业、保护生态，推动海西东北翼中心城市建设取得新成效，向新中国成立六十周年献礼。

2009年经济社会发展的主要预期目标是：地区生产总值增长12%左右，其中第一、第二、第三产业增加值分别增长5.5%、14.5%和12.5%；全社会固定资产投资增长18%；社会消费品零售总额增长18%；外贸出口增长10%；实际利用外商直接投资（验资口径）增长10%；财政总收入增长10%，地方财政收入增长9%；城镇居民人均可支配收入实际增长8%；农民人均纯收入实际增长8%；居民消费价格总水平涨幅控制在4%；城镇登记失业率控制在4%；人口自然增长率控制在8‰以内；单位生产总值能耗降低1.5%，化学需氧量排放下降3.2%，二氧化硫达标排放并努力控制排放量增长。在实际执行中，争取更好更快。

各位代表，去年省委常委会专题研究了《环三都澳区域发展规划》，提出“依法依规，规范运作；逐步开发，有序推进；科技创新，有效提升；安置保护，民生优先”的明确要求；9月30日，省政府正式批准实施《规划》。在这次代表大会上，还要对实施《规划》作出决议，把市委的决策通过法律程序，成为全市人民的意志。这是我市具有历史性、战略性、全局性意义的一件大事，标志着宁德经济社会发展进入了新阶段、掀开了新篇章。全市上下务必要按照市委二届五次全会的要求，把思想和行动切实统一到省委、省政府和市委的重大决策部署上来，把智慧和力量凝聚到加快推进环三都澳区域发展的工作大局上来，举全市之力、聚各方要素，扎扎实实地组织、运作、实施好《规划》，推动宁德加快发展、跨越崛起，努力打造海西东北翼中心城市。今年政府工作要紧紧围绕“保增长、扩内需、调结构、重民生”和环三都澳区域发展大局，重点抓好八个方面工作：

（一）着力农业增效、农民增收，扎实推进农村改革发展

加快农村体制机制改革。稳定和完善农村基本经营制度，保持现有土地承包关系稳定并长久不变。严格执行耕地保护和节约用地制度，坚守223万亩的耕地红线，确保基本农田总量不减少、用途不改变。加强土地承包经营权流转管理和服务，促进土地规模化经营。推进农村产权制度改革，巩固扩大集体林权制度改革成果，健全生态公益林管护补偿机制，完善林权抵押贷款等配套措施，深化小型农田水利设施制度改革，建立海域和内陆水域使用权利益补偿机制。深化农村金融体制改革，稳步开展农业政策性保险试点工作，加快村镇银行、小额贷款公司、农业担保公司和农村资金互助社组建步伐。完善党员干部驻村任职、农村“六大员”、科技特派员等工作机制，扶持农民专业合作社发展。

加快发展现代农业。加大农业科技投入与“五新”推广力度，促进科技成果转化应用。切实保障粮食安全，按照“稳面积、增产量、不抛荒”的要求，继续提高粮食收购最低保护价，逐步完善粮食直补、农资综合、农机具购置、良种等补贴办法，大力推广超级稻、再生稻，优化品种结构，提高单产水平，确保粮播面积稳定在210万亩、粮食产量稳定在69万吨以上；加强粮食储备管理、安全应急和市场供应体系建设，新建商品粮基地6000亩，确保粮食自给水平不下降。加快农业产业化进程，发挥“柘荣太子参”、“古田银耳”、“坦洋工夫”、“福鼎白茶”、“闽东大黄鱼”等品牌效应，大力实施“百龙带动”工程，力争建成农业生产示范区10个，产值超亿元的农业产业化龙头企业达10家。加强农业标准化和农产品质量安全工作，加快动植物检疫和农产品、食品质量安全检测检验等体系建设。办好第三届海峡两岸茶业博览会，结合闽东旅游文化、历史文化、畲族文化和特色产品的宣传推介，把茶博会办出特色、办出成效、办出水平；继续加强院市合作，扎实推进茶产业结构调整，提升茶叶精深加工水平，搞活茶叶流通环节，不断扩大闽东茶叶的知名度和影响力。科学修编水产养殖规划，调整优化水产业品种结构和养殖模式，大力发展滩涂、浅海生态健康养殖和深水网箱养殖，加快建设福建省水产品质量检测中心，力争水产品总产量达62万吨。大力推广食用菌工厂化生产新工艺和立体栽培技术，推进食用菌精深加工，力争食用菌鲜品产量突破60万吨。加快建设竹业富民工程和速生丰产林、名特优经济林、花卉基地，推进种植珍贵树种示范村建设。积极培育壮大畜牧、水果、蔬菜、药材、油茶等特色产业，促进农林牧渔业全面发展。

加快农村基础设施建设。完善农业基础设施，实施农业综合开发土地治理5万亩，完成26座中小型病险水库除险加固任务，加快福安茜安等大中型灌区续建配套和节水改造工程建设。提升农业防灾减灾能力，加强赛江、鳌江、霍童溪、七都溪、金溪、东湖等中小流域和城市内湖综合治理，加快重点县级城区防洪排涝工程建设，完成海堤除险加固六期工程，积极推进交溪流域上白石水利枢纽工程前期工作；抓好沙埕、三沙2个中心渔港和避风锚地建设。改善农村生产生活条件，解决13.4万农村人口安全饮水问题，新建一批农村公路“上衔下延”项目、危桥改造项目和农村客运站、农村候车亭，抓好15个乡镇435个村庄垃圾治理，新建8700口农村户用沼气池。

加快农村公共事业发展。巩固农村义务教育普及成果，抓好农村寄宿制学校宿舍建设和新增D级危房改造，提高农村义务教育学校教职工编制标准，解决好农民工子女就学和农村留守儿童教育问题。完善新型农村合作医疗制度，健全农村三级卫生服务网络，加快乡镇卫生院改革与发展，全面配齐乡镇卫生院基本诊疗设备，加强乡镇卫生院人才培养，用两年时间使全市乡镇卫生院基本达到国家建设标准。继续实施“一户一工一技能”工程，加强农村劳动力培训，做好农民转移就业和返乡创业服务，完成农村劳动力技能培训2.5万人，新增农村劳动力转移就业3.5万人。大力倡导移风易俗，进一步深化农村殡葬改革。深入开展经常性的科技、文化、卫生“三下乡”活动，推进城乡文化信息共享、乡镇综合文化站和村文化室、农村电影放映、农家书屋等文化惠民工程建设，丰富农民群众文化生活。

（二）全力扩大内需、拓展外需，确保经济持续较快增长

千方百计扩大投资拉动。今年全市安排重点建设项目100个，总投资1162.9亿元，年度计划投资162.1亿元，主要投向城乡基础设施、保障性安居工程、生态环境、社会事业以及产业结构调整和优化升级关键项目等领域。其中，在建项目75个，年度计划投资154.6亿元；预备项目25个，年度计划投资7.5亿元。集中力量突破项目前期，重点抓好衢宁铁路、京福高速铁路古田段及宁古支线、东南沿海货运铁路、沈海高速公路复线、京台高速公路（含宁古连接线、古屏连接线）、福泰高速公路、湾溪跨海大桥、三都岛跨海大桥以及连接主要港口作业区疏港铁路和疏港公路等重大基础设施项目前期工作，努力形成策划储备一批、前期推进一批、开工建设一批的良性循环。建成温福铁路宁德段，加快宁武高速公路、宁古二级公路、盐田至溪南疏港公路建设，动工建设滨海大道和白马作业区疏港铁路；开工建设城澳作业区1号、2号两个5万吨级多用途码头；加快福宁湾围海工程建设，动工建设后湾、大南塘围海工程；加快电网建设，建成220千伏福安韩阳、福鼎树兜输变电工程，动工建设220千伏福安溪尾、110千伏蕉城碧山输变电等工程。严格执行重点项目建设管理办法，加强项目的运作、协调和服务，按照项目建设程序，认真落实土地、环评、节能等管理要求，强化项目资金、质量、工期的稽查和审计，努力把重点项目建成精品工程。

千方百计推进消费持续增长。合理引导房地产消费。认真贯彻落实国家出台的促进房地产平稳健康发展的政策，调整公积金贷款政策，落实促进住房交易的税费优惠政策，鼓励发展二手房市场和住房租赁市场，支持居民购房；大力支持廉租住房、经济适用房、限价商品房及普通商品住房项目建设，着力解决城市低收入家庭住房困难。积极引导旅游消费。扎实做好宁德世界地质公园配套设施建设、环境整治、人文景观打造、地质科学研究、地质科普等工作，迎接联合国教科文组织专家评委的现场考评；加快推进福鼎市、屏南县争创中国优秀旅游城市，继续推进鲤鱼溪、九龙漈创建4A级景区；抓好旅游资源整合和要素配套，加强与周边城市的旅游协作和线路对接，加大旅游宣传促销力度，积极推行旅游招商，带动酒店业、会展业加快发展。积极培育汽车销售、教育培训、医疗保险、体育保健、拍卖租赁、担保服务等新的消费热点，满足城乡居民不同层次的消费需求。努力激活商贸流通。健全完善城乡商业网点和服务网络，动工建设电机电器、船舶修造物流配送基地和海峡大茶都二期工程，加快建设、改造、提升和完善食用菌、水产品、太子参等专业批发市场，为争取2010年举办海峡电机电器博览会、海峡水产品博览会做好准备；大力实施“双百市场工程”、“万村千乡市场工程”、“家电下乡工程”、“信服工程”、“农改超”工程和“便利消费进社区、便民服务进家庭”工程，鼓励连锁经营龙头企业向农村延伸，大力发展食品、日用品、药品、农资等经营点和加盟连锁“农家店”。切实加强市场监管。重点加强粮、油、肉、蛋、奶等基本生活必需品的生产、调运和储备，保障市场供给，保持物价稳定；继续深入开展产品质量和食品药品安全专项整治，加大治理“餐桌污染”，建设“食品放心工程”。

千方百计拓展对外贸易。认真落实国家和省、市出台的促进外经贸发展政策，在各县（市、区）设立外经贸发展专项资金，引导出口企业利用出口信用保险，降低出口收汇风险；大力实施“走出去”战略，支持企业境外办展和设立区域营销中心，扶持一批大宗传统特色产品出口和一批龙头生产型出口企业，带动优势产品扩大出口。鼓励具有自主知识产权和自主品牌产品出口，重点扶持电机、船舶、水产、食品等优势企业申报国家级、省级出口名牌，力争新增省级出口名牌企业10家；鼓励拥有自主品牌的企业以定牌生产方式，将生产制造环节向中小企业转移，实现品牌企业资源的有效整合，带动一批中小企业发展。建立和完善出口企业与金融、国税、外经贸、外管及口岸通关部门的联席会议制度，及时传递国际贸易中反倾销、反补贴和各种壁垒的最新动态，帮助企业解决出口中遇到的困难和问题。

（三）突出壮大规模、提质增效，增强工业经济整体实力

加快推进产业集聚。发展壮大临海产业集群。争取省政府正式批准设立海西宁德工业区，尽快组建工业区管理机构和合作开发公司，促成中海油公司启动项目建设；完成大唐火电二期工程并投产发电，抓好宁德核电一期、福安东元电机建设，积极推进大唐火电三期、大型钢铁、油气储备、福安镍合金等重大项目前期工作。做大做强传统产业集群。办好8个省级工业园区和19个市级工业集中区，进一步完善“飞地政策”，加快园区水、电、路、信息等基础设施和生活、教育配套设施建设，突出抓好已签约项目的落地建设和80项重点工业制造业项目，确保年内完成工业投资150亿元以上，亿元以上工业企业达到150家；加快电机电器、船舶修造、食品加工、汽摩配件等传统优势产业技术改造，力争电机电器产值突破150亿元。培育高新技术产业集群。完善东侨工业集中区扩展规划，加快推进聚合物锂离子电池等高新技术企业落地建设。

提升自主创新能力。扎实推进市高新技术创业服务中心和8个行业技术研发中心建设，继续实施“一县一业”星火专项、重点工业科研和企业技术开发项目，积极筹建国家级电机电器产品质量检测中心，提升省级化油器产品质量检测中心能力，建成省化油器行业技术开发基地。加快企业技术创新体系建设，建成20个市级企业技术中心。深化产学研联合，积极培育科技经纪企业和经纪人，抓好“6·18”项目对接和成果转化；充分发挥市政府院士、专家顾问的作用，以宁德市院士科技园为载体，吸引海内外具有较高学术水平和一定产业背景的科技领军人才来宁创业。加快培育国家级和区域性品牌，积极宣传推介“福安电机电器城”、“福鼎中国化油器城”等区域品牌和“泰格电机”、“安波电机”等企业品牌，力争年内新增工业类中国名牌2个。推进标准战略实施，积极组织和参与制修订4个国际标准和国家标准，鼓励企业采用行业、国家和国际标准，提高产品竞争力。

扶持工业企业发展。抓住中央、省加大扶持中小企业力度的机遇，认真研究政策、熟悉政策、用足政策，采取强有力措施，帮助中小企业摆脱困境、加快发展。建设小企业创业服务基地，开展福安、东侨等小企业创业基地试点工作，在融资担保、技术信息、人才培训、经营管理、法律咨询等方面提供支持，推动中小企业发展壮大。积极探索建立多种形式的中小企业信用担保机构，建立各县（市、区）中小企业信用担保机构风险补偿金，推动担保机构通过联合、兼并、重组以及吸收民间资金等多种方式增强实力。建立龙头企业资金链应急保障机制，完善工业企业运行服务网络，健全煤电油运和重要原辅材料调度机制，保障工业经济持续较快发展。

（四）强化区域联动、山海互动，统筹推进经济协调发展

加快环三专项规划编制。围绕《环三都澳区域发展规划》确定的功能定位和目标任务，加快专项规划和分区规划的编制。年内完成城市总体规划、岸线利用规划、土地利用规划、海域使用规划、环境保护规划、滩涂围垦规划以及水资源、能源、交通、林业等专项规划编制或修编，完成溪南、漳湾、赛江三个临海工业片区的控制性详规编制和环评工作。做好湿地红树林保护区、大黄鱼繁殖保护区和沿海防护林、生态公益林功能区划与《规划》的衔接工作。着手编制宁德港水域开放规划和航路指南，依法清理航道、锚地水域内的渔业设施等碍航物，加快推进口岸配套设施建设，争取上级有关部门批准宁德港海域扩大开放。继续争取国家各部委和省政府、省直各部门的支持，在与省口岸与海防办、福州海关、福建出入境检验检疫局、省公安边防总队、福建海事局签订支持环三都澳区域综合开发建设备忘录的基础上，促成更多省直部门与我市签订支持环三都澳区域发展协议。

增强中心城市综合功能。按照“临海、跨海、环海”三步跃升、打造绿色宜居海湾新城的目标，继续推进东侨新区提升拓展，加快推进漳湾、金涵等片区建设，实施三屿、铁基湾围海造地，延伸宁德中心城市南北临海两翼。加快城区路网建设，建成福宁路二期、火车站区域路网，动工建设学院路等城市道路。加强市政公用设施建设，加快推进五星级会展酒店、市垃圾焚烧处理厂、环东湖沿岸夜景和生态公园、城区生态防护及排涝工程建设。加强城市建设管理，强化城乡规划效能监察，继续深入开展城市环境综合整治活动和“清违”专项行动，为争创国家级园林城市和卫生城市打基础。

增强县域经济内在活力。各县（市、区）要按照市委二届五次全会确定的发展定位，在环三都澳区域发展大局中争作为、求先行，不断提高县域经济实力。蕉城、东侨要壮龙头、做表率、求先行，充分发挥中心城市建设的主力军作用，集中精力做大产业规模、提升科技含量、做优城市环境、做强经济实力；福安要挑大梁、走前头、求先行，充分发挥产业基础较好、民间活力较强的领跑者作用，集中精力打造临海港口工业城市，争取尽快跨入全省经济十强县，进而跻身全国百强县；福鼎要当先锋、谋对接、求先行，充分发挥对接浙南的桥头堡作用，着力打造沿海生态型工业城市；霞浦要立潮头、争跨越、求先行，充分发挥吸聚临海大工业的主阵地作用，着力打造临海重化产业聚集的新增长极；古田、屏南、周宁、寿宁、柘荣要强特色、重互动、求先行，充分发挥支持临海率先突破的大腹地作用，不断培育形成加快发展的特色亮点，联手打造沿海开发的“后花园”，努力形成与沿海一线优势互补、遥相呼应的发展格局。

（五）狠抓节能减排、环境保护，致力建设生态文明

抓好重点领域节能。突出抓好冶金、化工、电力、建材、制革等重点行业和年耗能1000吨标准煤以上企业的节能工作，全面关闭“十五小”、“新五小”企业。重视发展和推广应用建筑节能新技术、新材料和新产品。加快淘汰老旧高耗能交通工具，引进和推广车用代用燃料和清洁燃料汽车，积极推广节油新技术。抓好机关单位的节水、节电、节约办公用品等工作。推进商业和民用节能，积极推广照明、制冷、供热等方面的节能新技术、新产品。加快淘汰、更新落后的农业机械和渔船设备，推进以沼气为重点的农村能源工程建设，大力推广利用太阳能。

严格控制污染排放。严格执行建设项目环境影响评价制度和环保“三同时”制度，建立有力的源头防控体系。继续依法整治敖江古田段、闽江古田段、赛江（交溪）、霍童溪和全市县级以上湖库型饮用水源保护区。加快推进城市污水、垃圾处理设施建设，建成福安、古田、柘荣污水处理厂，确保2010年前，全市所有县（市）都建成投产一座以上污水处理厂和垃圾处理厂。深入开展环保专项行动，加大船舶拆解、石板材、小钢铁、制革、造纸等重点行业污染治理力度，加快处理历年省市挂牌督办环境案件；集中整治工业园区环境违法问题，加快建设污染集中治理设施，坚决纠正降低环保准入条件、阻碍环境执法、降低或取消排污费等违法行为。

科学开发保护资源。合理整合和利用岸线资源，认真实施沿海港口布局规划，实行最严格的管治措施，切实管好港口岸线、前沿海域和后方陆域。加强海洋环境监测，合理调整养殖区范围，加大清理污染大、效益低的浅水养殖，努力减少冰鲜饵料投放，积极发展深水大网箱养殖，有效改善海域生态环境。加强生态示范区、风景名胜区建设和自然保护区管理，抓好高速公路、铁路、国道、省道和主要流域两侧一重山等重点区域的生态保护，推进生态公益林、湿地、沿海防护林、天然阔叶林的保护和建设，强化森林火灾防控和病虫害防治，加大植被防护力度，有效防治水土流失，促进生态修复。

（六）持续深化改革、扩大开放，不断激发发展活力

扎实推进关键环节和重点领域改革。深化行政管理体制改革，推进以转变政府职能为核心的地方政府机构改革，继续深化乡镇机构改革试点，深入开展议事协调机构和非常设机构清理规范工作，推进事业单位分类改革；继续做好行政审批事项清理工作，全面推行网上审批。强化国有资产监管，推进内贸企业改制，完善国有资本经营预算管理、业绩考核、激励和约束等机制，实现国有资产保值增值。深化医药卫生、教育、科技、文化等社会领域体制改革。抓好资源性产品价格改革，落实好国家燃油税费、天然气价格形成机制改革，实施水资源费征收使用管理办法，完善污水、垃圾处理的收费政策，促进公共资源市场化配置。

持续提升利用外资水平。完善“6·16”宁德投洽会等重要平台功能，积极组团参加境内外重大招商活动，推出一批城建、港建、交通、环保、供水、燃汽等基础设施项目对外招商，引进一批临海重化、能源项目、物流企业落户环三都澳区域，吸引更多战略投资者投资造船、电机、汽摩配件、农产品精深加工等传统优势产业。坚持项目招商与产业发展、园区建设相结合，引进投资大、科技含量高、综合效益好和产业链缺失环节的项目，促进关联产业加快聚集。认真落实签约项目跟踪落实工作机制，进一步提高项目报批率、动工率和到资率。

切实加强区域经济协作。积极主动对接长三角，重点加强与浙南地区、赣东北地区的区域合作，大力推进金融、物流服务、商贸流通、旅游开发、基础设施及招商平台建设等领域的双向对接，主动承接其产业、资金、技术和人才转移。继续深化福州与宁德山海协作以及闽东北五地市、四省十四市的区域合作，坚持政府引导、市场导向、企业主体相结合的协作模式，努力在广泛交流协作中获取商机、引进项目。加强与港澳地区的经贸合作，增进与海外侨胞、工商界企业人士的联络和沟通，大力维护归侨侨眷、海外侨胞合法权益，吸引港澳同胞、海外侨胞到我市投资兴业。

积极扩大宁台交流合作。抓住两岸和平发展的有利时机，加快建设对台直航码头及配套通关设施，开通宁德港与基隆港的双向航线，推进宁马台客货运直航常态化。推动我市与基隆市在港口经济、会展、渔业、旅游等方面的合作和人员、文化交流。加快规划建设台湾高科技工业园区和台湾农民创业园区，打造一批台商创业载体，拓展先行先试平台。强化食品、生物制药、电机等优势产业的产业链招商，积极承接石化、钢铁、机械、光电、电子信息等产业转移，力争更多的台资项目落户宁德。加强对台农业合作，引导台商投资我市农业综合开发、农产品精深加工等产业。打造“闽东北亲水游”旅游品牌，主动与台湾共享旅游资源，互推旅游市场，实现宁台旅游深度对接。加快台湾水产品集散中心建设，争取批准设立海峡两岸（宁德）名优特商品交易中心，提升对台小额贸易规模。完善台胞接待站、台轮停泊点功能，推进输台渔工劳务派遣工作。充分发挥圆瑛法师、陈靖姑、甘国宝等民俗文化优势，积极推动两岸往来和民间交流，吸引更多台湾同胞来宁寻根谒祖、探亲访友。

（七）切实加强运作、突破瓶颈，努力拓展发展空间

严格加强财政收支管理。面对严峻的财政形势，坚持把财政工作重点放在保工资、保运转、保重点上，确保财政正常运转。积极培育财源，综合运用贴息、奖励、补助等财政手段，支持符合国家产业政策和市场准入条件的项目建设，支持房地产、旅游等第三产业发展，进一步巩固传统优势财源，努力挖掘新增财源。密切关注国家宏观政策对财政收入的影响，及时准确把握税源，加大征收力度，做到依法征收、应收尽收，确保财政收入持续有效增长。坚持“过紧日子”和“勤俭办一切事业”的方针，按照“量入为出，量力而行”的原则，调整财政支出结构，严格控制和减少一般性财政支出，节约公用经费支出；强化财政预算刚性约束，防止新开增支口子。继续深化财政改革，强化部门综合预算，完善国库集中支付制度，推进行政事业单位固定资产动态监控；统筹预算内外财力，强化预算外资金管理，增强政府宏观调控能力；强化政府投资项目管理，试行政府投资项目公示和后评估制度，提高项目概预算的准确性，严格投资控制和稽查审计，切实提高政府性资金的使用效益。

大力拓宽筹资融资渠道。加强与开发性银行、进出口银行及商业银行的沟通、合作，积极争取银行信贷额度，多渠道筹措建设资金。加强政银企协作，引导金融机构加大对重点建设、中小企业、“三农”工作、社会就业、技术改造、节能减排的信贷支持。积极筹建三都澳农村商业银行，多渠道筹措环三都澳区域开发建设资金。做大做强国投公司，通过发行项目建设债券、公司债券，做大融资主体，增强融资功能。积极鼓励和启动民间资本和外资投入，最大限度放宽民间投资准入领域，引导民资和外资参与重点工程、重点项目建设。进一步落实扶持民营经济发展的政策措施，定期发布产业政策导向和项目指引，推动民间资本与重点产业、基础设施、社会事业和现代服务业深度对接。

依法突破征地拆迁难题。严格依法开展征地拆迁，切实把思想工作做在前，把相关程序做到位，把具体任务落实到相关的每个乡镇、每个村、每一户、每块地，做到任务明确、责任具体、人员到位，确保各项征地拆迁任务按时完成。建立健全征地拆迁补偿机制，合理确定征地拆迁补偿标准，落实被征地农民社会保障制度，切实保护被征地拆迁群众的合法权益。积极加强与上级业务部门的联系，千方百计争取用地指标，特别是重大项目专项用地指标。做好内部挖潜文章，按照科学发展的要求，通过围海造地、山地利用，增加建设用地资源。加大土地清理力度，对征而未用、多征少用、土地闲置、投资强度未达到要求的，依法坚决征收土地闲置费或收回土地使用权。

（八）坚持着力民心、着力民生，积极构建和谐社会

持续办好惠民实事。继续集中财力，实施一批为民办实事项目。加强城乡就业保障工作，重点帮助城镇零就业家庭、农村贫困家庭、就业困难人员、高校毕业生和退役军人就业，新增城镇就业2.4万人、下岗失业人员再就业6000人。继续完善社会保障体系，扩大养老、失业、医疗、工伤、生育保险覆盖面，提高农民工、灵活就业人员、被征地征海农民、城乡非就业人员等群体的参保率和保障水平。强化社保基金监管，实现保值增值。适当提高城乡居民最低生活保障标准和最低工资标准，完善企业职工工资正常增长机制和支付机制。加强社会救助体系建设，积极发展社会福利、社会慈善事业，扎实做好离退休职工、孤老户、残疾人、重点优抚对象和革命“五老”人员的帮抚工作，提高优抚对象抚恤和生活补助标准。加快建设市社会福利中心，动工建设2座县级社会福利中心，新建和改扩建农村敬老院11所。继续开展老区工作优先行动，认真落实库区移民后期扶持政策，支持革命老区、少数民族地区、海岛等经济欠发达地区和移民安置区基础设施建设。完成“造福工程”和地质灾害险情村搬迁2.5万人。加快对口支援地震灾区援建项目建设，积极开展劳务合作、支医支教等对口支援活动，切实帮助灾区人民加快重建步伐。

协调发展社会事业。根据城市和产业发展趋势，按照小学就近入学、初中相对集中的原则，合理布局城区中小学校，优化配置教育资源；全面实行城乡免费义务教育，完善家庭经济困难学生资助制度，免费为义务教育阶段学生提供教科书，补助农村义务教育阶段寄宿生生活费，建立低保家庭普通高中学生助学金制度，减免中职学校低保家庭学生学费，免除特教学校学前阶段、高中阶段残疾学生学费；加强中小学校标准化建设，实施中小学校舍抗震安全加固改造工程；深化普通高中改革，加快普及高中阶段教育；整合市级职业教育资源，加强县级职教中心建设，抓好中等职业学校教学实训基地建设；进一步加快宁德学院和宁德职业技术学院建设；开展早期教育，重视学前教育，关心特殊教育和老年教育，发展现代远程教育和社区教育，规范民办教育；加强教师队伍建设，稳步推进中小学教师绩效工资制度改革。加强公共文化服务体系建设，推进图书馆、博物馆等公共文化基础设施前期工作；加快发展文化产业，扶持壮大文化骨干企业；加强群众性文化建设，积极组织开展内容健康向上、形式多姿多彩、群众踊跃参与的文化活动；继续做好“中国木拱廊屋桥传统营造技艺”申报急需保护的世界非物质文化遗产名录工作，争取成功申报闽东畲族文化生态保护区；深入开展第三次全国文物普查，做好重点文物保护、文化典籍整理和保护以及非物质文化遗产的传承和保护工作；保护和发展闽东畲族文化、茶文化、廊桥文化、红土地文化等地方特色文化，打造一批代表宁德形象的文化品牌。加快市体育中心二期工程建设，继续实施全民健身和农民体育健身工程，办好宁德市第三届运动会。完善公共卫生体系建设，加强重大疾病的预防和控制，深入开展中小企业危险化学品职业病危害、餐饮业卫生、学校卫生等专项整治，提高应对突发公共卫生事件能力；着力推进城镇职工基本医疗保险、城镇居民基本医疗保险、新型农村合作医疗和城乡医疗救助制度全覆盖，力争应保尽保；积极稳妥推进城乡医疗卫生管理体制改革，加快社区卫生服务站标准化建设。深入开展创建人口计生优质服务先进县（市、区）活动，稳定低生育水平，治理性别比偏高问题，提高出生人口素质。

维护社会安定稳定。深入开展平安创建活动，加强社会治安综合治理，完善社会治安防控体系，创新城乡社区警务工作机制，依法防范和打击违法犯罪活动。健全矛盾排查调处机制和社会利益协调机制，依法妥善处置重点信访、越级上访、老信访问题和群体性事件，努力把不稳定因素化解在基层、化解在萌芽状态。严格落实安全生产责任制，强化安全生产监管监察，重点抓好道路交通、非煤矿山、危险化学品、消防、建筑施工、水上交通与渔业船舶、特种设备、民爆器材与烟花爆竹、旅游、学校等重点行业、重点领域的专项整治，全面排查治理安全隐患，遏制各类较大事故。强化应急管理工作，提高突发公共事件信息报送的时效和质量，及时有效地处置好各类突发公共事件。

加强民主法制建设。认真贯彻落实监督法及我省实施办法，坚持向市人大及其常委会报告工作制度，自觉接受市人大及其常委会的法律监督、工作监督，依法执行人大及其常委会的决议决定，认真办理人大代表建议、意见，积极支持人大代表开展视察活动。坚持定期向市政协通报情况，主动接受市政协的民主监督，认真办理政协委员提案，虚心听取各民主党派、工商联、无党派人士和各界人士的意见。充分发挥工会、共青团、妇联等人民团体的桥梁和纽带作用，主动接受新闻舆论和社会公众监督。大力发展社会主义民主，依法落实人民群众的知情权、参与权、表达权、监督权，切实保障人民群众的民主权利。

推进精神文明建设。加强公民道德建设和青少年思想道德教育，大力弘扬闽东精神，扎实开展群众性精神文明创建活动，积极倡导志愿服务。深化双拥共建，健全国防动员体系，加强民兵预备役部队建设和人防、海防、优抚安置等工作，巩固发展军政军民团结。贯彻落实党的民族、宗教政策，深入开展民族团结进步活动，依法管理宗教事务。高度重视老干部、老龄和残疾人事业，认真做好新形势下的侨务、对台、外事等工作，继续抓好人事、编制、审计、统计、档案、地震、地方志、水文气象、关心下一代等工作。

三、致力先行必须建设对人民负责的政府

新的形势、新的任务，对今年政府工作提出了新的更高要求。各级政府要认真贯彻落实市委的决策部署，勤勉尽责，积极作为，力求先行，努力建设对人民负责的政府。

（一）在开拓创新中增强先行意识。开拓创新是加快先行的不竭动力，在开拓创新中才能不断拓展作为空间，激发先行活力。要解放思想谋先行，在思想上与先行的要求相对接，从落后的思想观念中解放出来，以思想的新解放引领先行发展的新高潮。增强信心争先行，积极应对困难和挑战，立足自身，锐意进取，同心协力，共克时艰，努力从各种不利因素的倒逼中开创发展新天地。注重谋划求先行，切实把握当前的宏观形势和发展趋势，提高决策的科学性，增强措施的协调性，真正把政策化为生产力，把机遇变为现实发展成果。积极实践促先行，切实把学习实践科学发展观融入到推动环三都澳区域发展的进程中，落实到今年各项目标任务上，持续举措，持续效应，持续发展，积极拓展先行的实践，以实实在在的工作业绩推动先行发展。

（二）在持续运作中力求先行实效。持续运作是推进先行的重要抓手。要明确持续运作的要点，落实持续运作的举措，努力实现政策决策的最大成效。坚持快节奏推进，强化时间观念、效率观念，发扬苦干精神、实干精神，真正把心思放在工作上、精力用在落实上，进一步加快运转速度、提高工作效能，确保各项目标任务按时完成。坚持强力度攻坚，广大干部特别是主要领导干部要勇于承担责任，敢于触及矛盾，善于深挖症结，以强有力的措施突破难题、化解矛盾、推动工作。坚持高效率落实，认真执行目标责任制，对照市委、市政府确定的目标任务，对每项工作进行细化、量化，明确责任人，列出时间表，一项一项抓好落实。今年，市政府将继续与各县（市、区）政府签订重点专项工作责任状，年底对执行责任状情况进行绩效考评，兑现奖惩。

（三）在勤政廉政中推动先行发展。勤政廉政是保障先行的内在要求。要坚持科学理政，严格执行民主集中制，认真贯彻实施政府信息公开条例，大力推进决策科学化、民主化，增强决策的透明度、参与度和信任度；深化政府绩效评估，完善评估体系，运用评估结果，促进政府及其工作人员认真履职尽责；大力弘扬求真务实精神，加强政策研究工作，对上级的决策部署，花更多的心思研究，用更多的时间推进，力戒空谈，务求实效。坚持依法行政，落实依法行政实施纲要，严格依照法定权限和程序行使权力、履行职责，提高政府公信力；规范行政服务中心运作，所有进入中心的审批项目必须充分授权，收办件全程在中心内完成。坚持廉洁从政，认真落实党风廉政建设责任制，强化对重点部门、重点事项和重点岗位的监督，让权力在阳光下运行，进一步树立为民、务实、清廉的良好形象。

各位代表，加快宁德发展，实现宁德崛起，是全市人民的共同愿望，是社会各界的殷切期待。让我们紧密团结在以胡锦涛同志为总书记的党中央周围，在省委、省政府和市委的坚强领导下，进一步坚定信心、迎难而上，有效作为、力求先行，全力推进环三都澳区域发展和海西东北翼中心城市建设，为开创宁德更加美好的明天而努力奋斗!
